# Betulin-3,28-diphosphate. Physico-Chemical Properties and In Vitro Biological Activity Experiments

**DOI:** 10.3390/molecules23051175

**Published:** 2018-05-14

**Authors:** Nina B. Melnikova, Darina S. Malygina, Irina N. Klabukova, Denis V. Belov, Viktor A. Vasin, Pavel S. Petrov, Alexander V. Knyazev, Alexey V. Markin

**Affiliations:** 1 Department of Pharmaceutical Chemistry, Privolzhsky Research Medical University, Minin sq., 10/1, 603950 Nizhny Novgorod, Russia; mds73@yandex.ru (D.S.M.); klabukova.2054@mail.ru (I.N.K.); belov.denbel2013@yandex.ru (D.V.B.); 2 Department of Chemistry, N.P. Ogarev Mordovian State University, Bolshevistskaya St. 68, 430005 Saransk, Russia; vasin@mrsu.ru (V.A.V.); petrovps@phys-chem.mrsu.ru (P.S.P.); 3 Department of Chemistry, Lobachevsky University, 23/5 Gagarin Av., 603950 Nizhny Novgorod, Russia; knyazevav@gmail.com (A.V.K.); markin@calorimetry-center.ru (A.V.M.)

**Keywords:** betulin-3,28-diphosphate and its sodium salt, thermoanalytical properties, antioxidant activity

## Abstract

Betulin-3,28-diphosphate (BDP) obtained by phosphorylation of betulin using POCl_3_ has two main structural forms—BDP-1 and BDP-2—which differ in ethanol solubility, melting point, FTIR spectra, thermoanalytical characteristics and biological activity. Betulin-3,28-diphosphate and its sodium salt (Na-BDP) were characterized using ^13^C and ^31^P-NMR spectra, powder XRD experiments, as well as differential scanning calorimetry (DSC) and thermogravimetric analysis (TG) methods. The exo-effects at 193 ± 8 °C for ethanol soluble BDP-1 samples (−19.7 ± 0.2 kJ∙mol^−1^) were about three times less than for ethanol insoluble BDP-2 samples f (−70.5 ± 0.7 kJ∙mol^−1^). The DSC curves of Na-BDP-1 and Na-BDP-2 characterized the endo-effects having a maximum at 95–112 °C. Water-soluble Na-BDP-1 was obtained as needle-like crystals, unlike poorly crystalline Na-BDP-2, whereas BDP-1 and BDP-2 aged with time and were isolated as amorphous substances. In vitro experiments on rats showed that compared to the control, Na-BDP-1 increased catalase and SOD activity and improved energy metabolism more effectively than Na-BDP-2.

## 1. Introduction

Lupane-type triterpenoids such as betulinic and betulonic acids, betulin succinates, betulin acetates and other esters of organic acids, show antitumor, lipid-lowering, hepatoprotective and antiviral properties [1,2,3,4,5]. The therapeutic use of betulin derivatives is limited to their poor solubility (from 1 to 100 μg∙L^−1^) and accordingly low bioavailability.

One of the ways to improve bioavailability of betulin derivatives as the potential active pharmaceutical ingredients is the synthesis of their derivatives containing sulfate, sulfonate, phosphate and phosphonate groups [6,7,8]. The derivatives of phosphoric acid have advantages, because their biotransformation easily proceeds under endogenous phosphatases, phosphorylases and kinases of blood and liver [9].

Betulin-3,28-diphosphate has proved to be a broad-spectrum drug in vitro and in vivo studies [8,10,11,12]. It was shown that betulin-3,28-diphosphate can be an antibacterial agent [10], an effective antiviral and antifungal agent [8,11] as well as an inhibitor of complement system activation, which plays an important role in the treatment of many systemic diseases [12].

Although the biological activity of betulin-3,28-diphosphate is well-known, its physicochemical properties which determine solubility, spectral data, physicochemical constants, impurity profile of the compound have not been studied yet. Along with the existing advanced analytical methods for chemical characterization (i.e., fingerprinting) of potential medicines, the need for more rigorous evidence of their authenticity is growing.

Every medicine synthesis and its purification result in a characteristic imprint of a substance. In this context, different structural modifications contain important information on the synthesis history of substances. Considering that the information regarding the synthesis of these substances is often confidential, it is necessary to include a technique that will allow comprehensive chemical characterization of the substances.

Thermoanalytical methods (DSC/TG) are among the most important techniques to take physicochemical measurements and determine multiple structural forms and their phase transitions of a singular chemical entity [13,14,15,16,17,18,19]. It has been shown earlier that DSC can reliably characterize the observed phase transition with <5 mg of a tripeptide sample [20].

The information on DSC/TG characteristics can reveal a unique BDP pattern in the used synthesis and purification. The combination of thermoanalytical methods and other analyses has already been employed to study solid-state interactions of medicines with pharmaceutical excipients [21], but there are no available data in the literature on BDP analysis. Thermoanalytical methods were used to study betulin and betulinic acid polymorphism and solvatomorphism [19]. Solvates with ethanol and DMSO have not only different parameters of crystal lattices, different solubility, but also different pharmacological activity.

In this work we developed a synthesis of betulin-3,28-diphosphate (3β,28-diphosphate- lup-20(29)-ene, BDP) and studied physicochemical properties and its sodium salt using potentiometry, DSC/TG, FTIR and powder XRD analysis. The final goal was to obtain BDP or its Na-BDP salt having good solubility and reproducible antioxidant activity results.

**Scheme 1:**
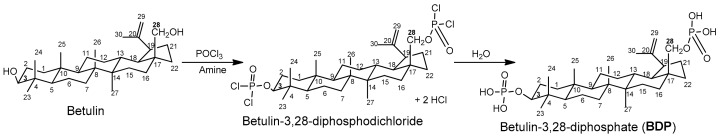
Synthesis of betulin-3,28-diphosphate.

BDP was synthesized by a method similar to the one used to obtain steroid phosphates [22], using betulin and phosphorus oxytrichloride (POCl_3_) in an organic solvent in the presence of triethylamine or pyridine as HCl acceptors (Scheme 1).

## 2. Results and Discussion

### 2.1. Properties of the Betulin-3,28-diphosphate

The solubility, melting point, and FTIR spectra of betulin-3,28-diphosphate (BDP) samples having the same chemical composition depended on the methods of its synthesis, purification and storage conditions. The first group of BDP samples (BDP-1) were readily soluble in ethanol (up to 10%) and had a melting temperature equal to 147–148 °C (*n* = 20). The second group of BDP samples (BDP-2) were limitedly soluble in ethanol (up to 1%) with a melting temperature equal to 156–160 °C (*n* = 15). The differences of BDP-2 sample properties were not due to the presence of monophoshorylated compounds, because the ^31^P-NMR spectra of BDP-2 samples were the same as the ^31^P-NMR spectra of BDP-1 samples and contained only the two signals that are characteristic for the C-O-P fragments at C-3 and C-28 for both BDP-1 and BDP-2 (Figures S1 and S2), and the integral ratios of these signals were 1:1 in all cases. The BDP structural forms in DMSO-d_6_ solution both in the presence and in absence of H_3_PO_4_ (Ph_3_P or Met_3_P standards) had similar ^31^P-NMR spectra (Figure S2). Control over hydrolysis products was exercised by adding H_3_PO_4_. Two phosphorus signals corresponding to diphosphate esters of betulin were observed, the integral ratio of signals being 1:1. Figure S2b shows the signal of a Ph_3_P standard (δ = −5.93 ppm). Figures S1 and S2b show the H_3_PO_4_ signal. Moreover, phosphorus content was the same for BDP-1 and BDP-2 samples after drying. Such different properties can be explained by the greater ability of the betulin derivatives dimers of BDP-1 to include the solvent (ethanol, water, dioxane) in their structure [19].

The boiling water treatment, reprecipitation by acetone, which was not included in the betulin structure and the action of an aqueous solution of HCl on sodium salt of BDP were applied to remove the solvent molecules from the BDP dimers of the first group.

**Figure 1:**
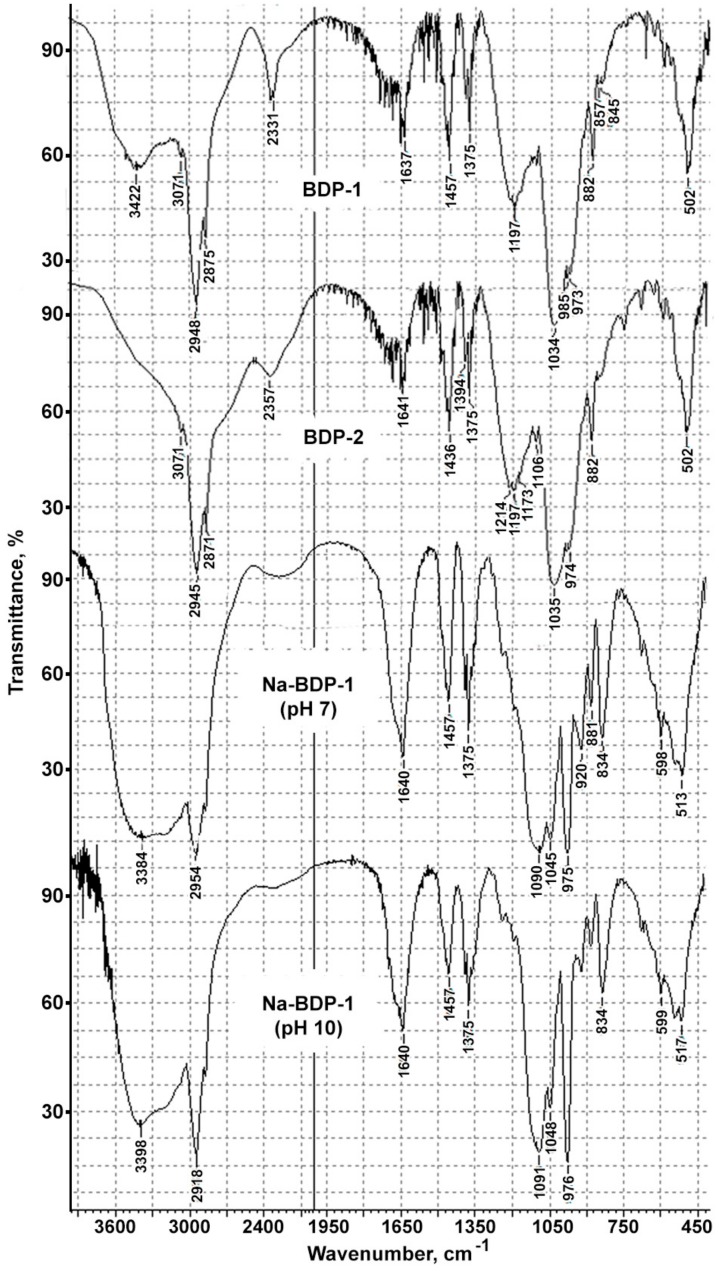
FTIR spectra BDP and Na-BDP: (**a**) Highly soluble in ethanol BDP-1; (**b**) BDP-2 sample obtained by acetone deposition from ethanol solution; (**c**) Na-BDP-1 formed at pH 7; (**d**) Na-BDP-1 formed at pH 10. FTIR was measured twice for 20 samples of each kind of BDP and Na-BDP.

The FTIR spectra of BDP samples having different solubility and melting temperature differed in the “fingerprint”region, and also in the region corresponding to the stretching vibrations of phosphoryl-POH comb (2331, 2342, 1740–1650 cm^−1^ br. w.), P-O st, P=O st (1216–1196, 1100–940 cm^−1^), and hydroxyl groups—3400–3300 cm^−1^ (Figure 1a,b).

Consequently, BDP samples having differences in FTIR spectra, melting temperature and solubility due to intermolecular interactions between the molecules of compounds or the molecules of the solvent and compound, may be considered as different structural forms of BDP [16].

**Figure 2:**
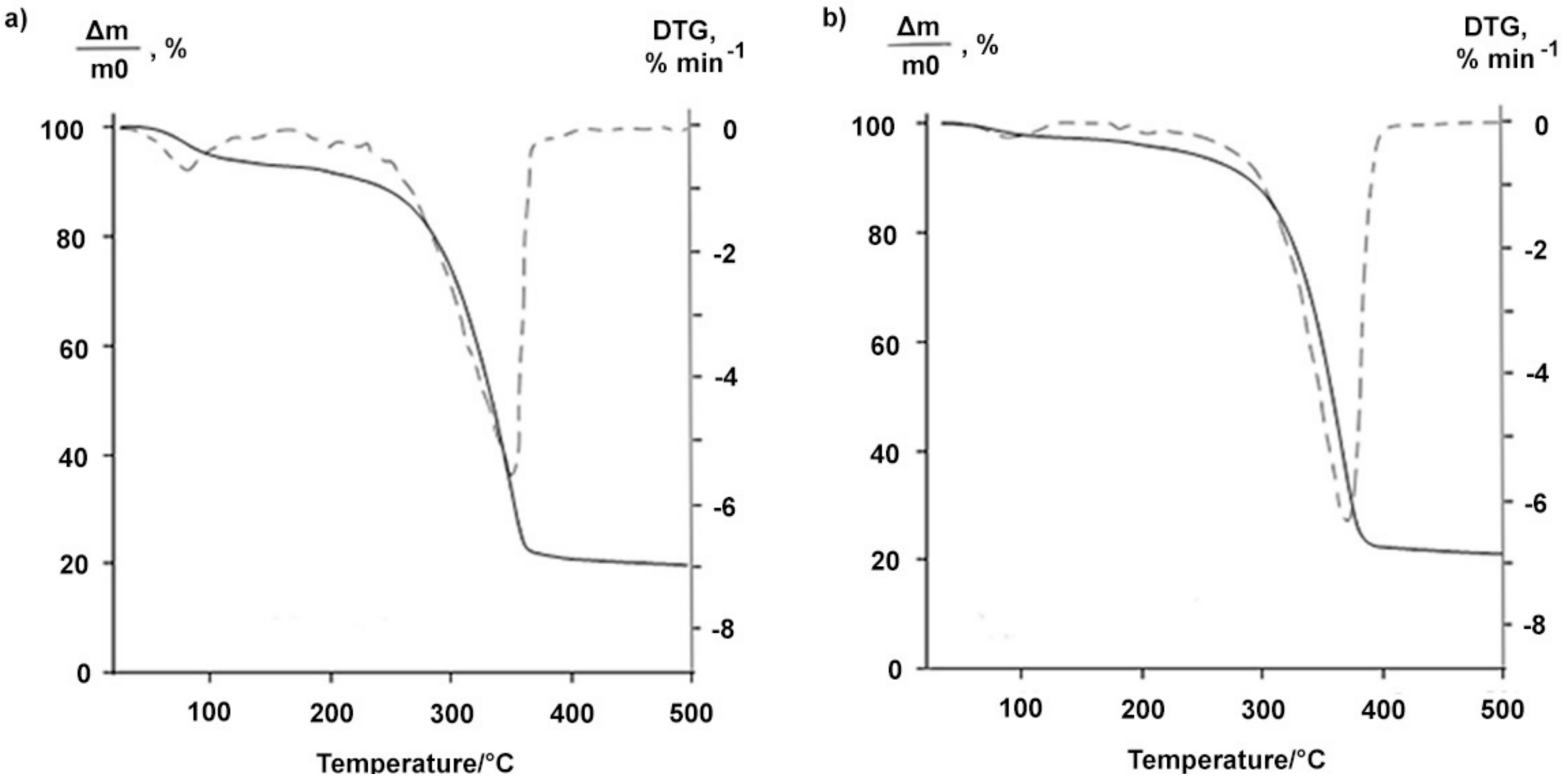
TG (solid line) and DTG (dashed line) curves of the BDP-1 (**a**) and BDP-2 (**b**).

The BDP forms had different thermoanalytical characteristics. The TG curve of highly soluble BDP-1 in ethanol had a stage characterizing the mass loss equal to 7% within the temperature range 72–150 °C (Figure 2a). The fact is likely to correspond to the removal of the solvent from the BDP samples. In contrast, the TG curve of the sample BDP-2 obtained by precipitating acetone from the ethanol solution did not have a stage in this temperature range indicating the absence of solvent molecules in the sample (Figure 2b).

**Figure 3:**
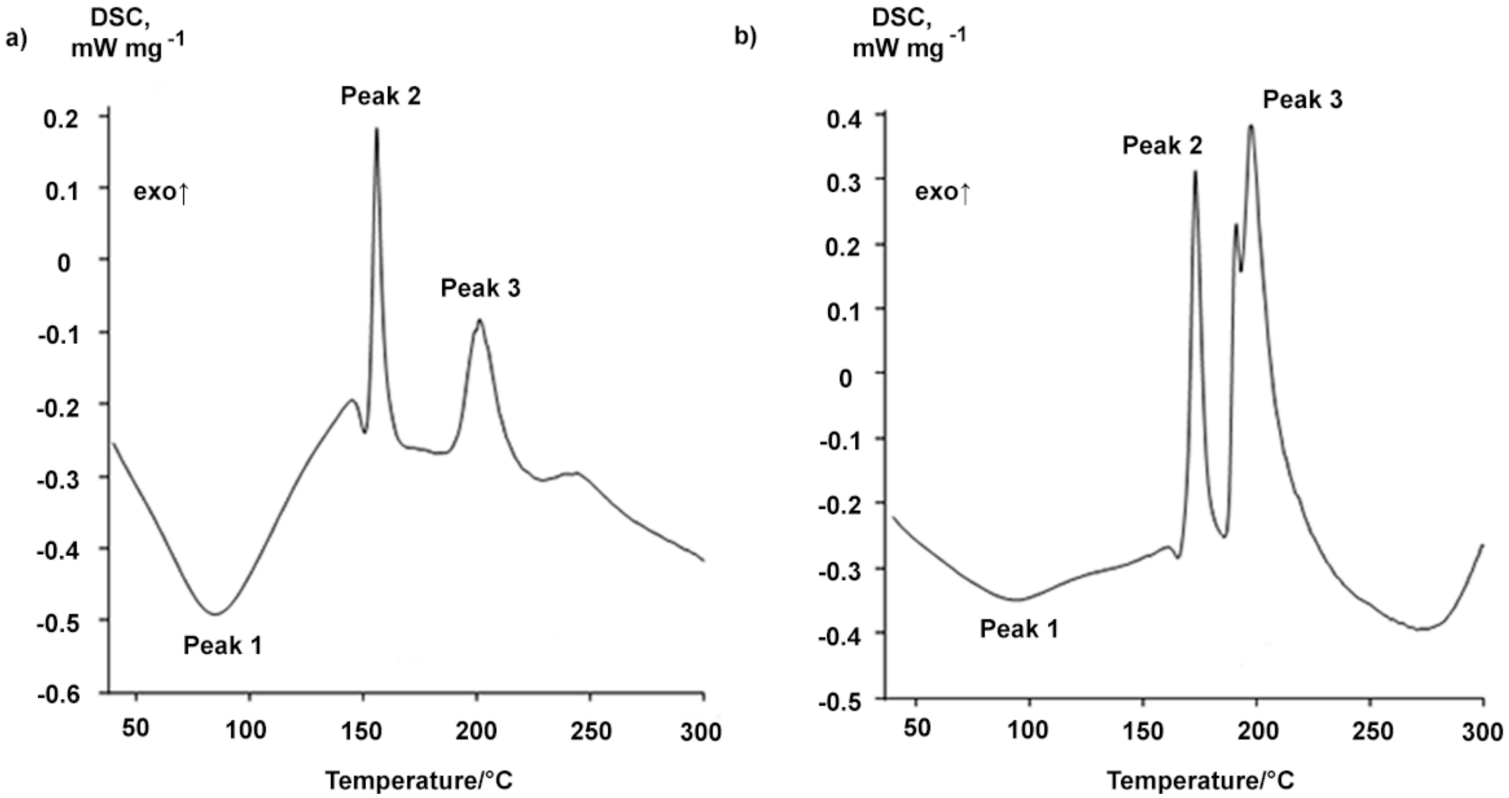
DSC-curves of the BDP-1 (**a**) and BDP-2 (**b**).

The BDP samples were analyzed in more detail using differential scanning calorimetry (DSC). The DSC profile of BDP-1 shows two main peaks characterizing different exothermic processes. The first process proceeds within 151.2–155.9 °C, ΔH_1_ = −15.5 ± 0.2 kJ∙mol^−1^; the second one is observed within 183.5–200.8 °C, ΔH_2_ = −19.7 ± 0.2 kJ∙mol^−1^ (Figure 3a, Table 1). Moreover, a wide fuzzy peak (peak 1) appeared at 70–90 °C on the DSC curves (Figure 3a). This can be explained by either the removal of solvent molecules from the probable BDP inclusion complexes or by glass transition in the BDP samples.

The exo-effects of BDP-2 reprecipitated by acetone from BDP ethanol solution are observed within 161.3–172.8 °C, ΔH_1_ = −23.5 ± 0.2 kJ∙mol^−1^; and 185.9–197.3 °C, ΔH_2_ = −70.5 ± 0.7 kJ∙mol^−1^ (Figure 3b, Table 1).

In general, for all BDP samples, the maximum of the first peak on DSC curves of the exo-effect varied within 150 ± 13 °C, and the second peak maximum varied within 193 ± 8 °C (Table 1). The enthalpy ΔH of the first exo-effect (peak 2) for all samples varied from −10.0 to −23.5 kJ∙mol^−1^, and the ΔH value of the second exo-effect (peak 3) strongly depended on the structural type. The ΔH value equaled to −19.7 ± 0.2 kJ∙mol^−1^ for BDP-1, and the ΔH exo-effect of BDP-2 varied from −67.1 to −80.4 kJ∙mol^−1^ (Table 1).

These findings enable to use the value of the enthalpy of the second exo-effect as well as the solubility and melting temperature, as a distinctive feature of BDP structural forms. It is probable that two or three-fold increase in the enthalpy of the second exo-effect for different BDP samples due to topochemical processes in solid state.

**Figure 4:**
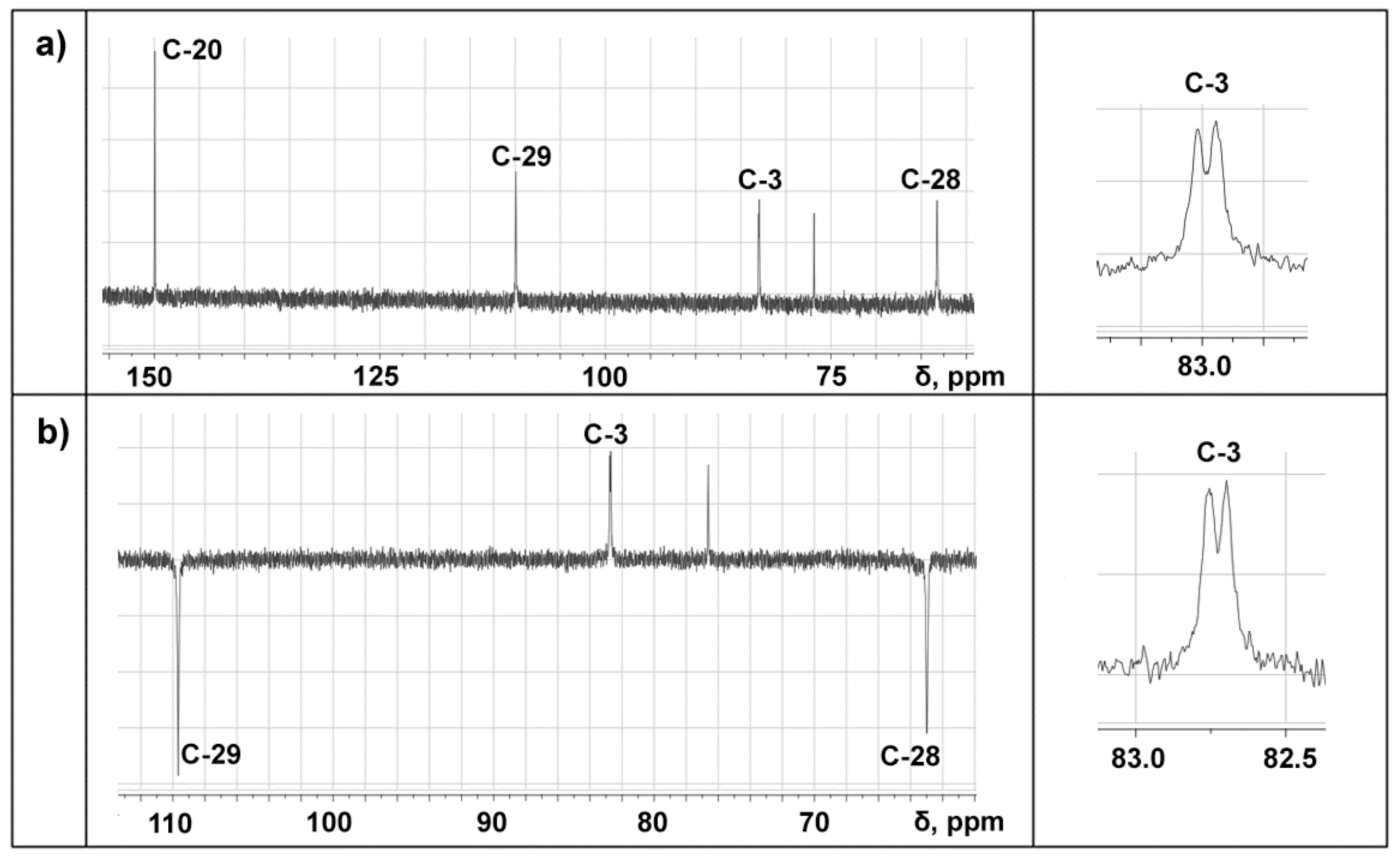
^13^C-NMR spectrum (**a**) and dept spectrum (**b**) of BDP-1 (DMSO-d_6_, TMS standard).

Besides, topochemical process may be the transition of the labile α-epimer at C3-atom of **BDP** to the β-epimer in their mixture. These epimers transitions can be studied using NMR spectroscopy, since the α- and β-epimers of betulin derivatives, e.g., betulinic acid, display differences in their spectra, but the differences are insignificant. Two BDP C3-atom signals having a difference equal to 0.06 ppm (6.25 Hz) are observed in the ^13^C-NMR and ^13^C-DEPT spectra of BDP-1, while such splitting is absent in the spectra of BDP-2 (Figure 4).

**Figure 5:**
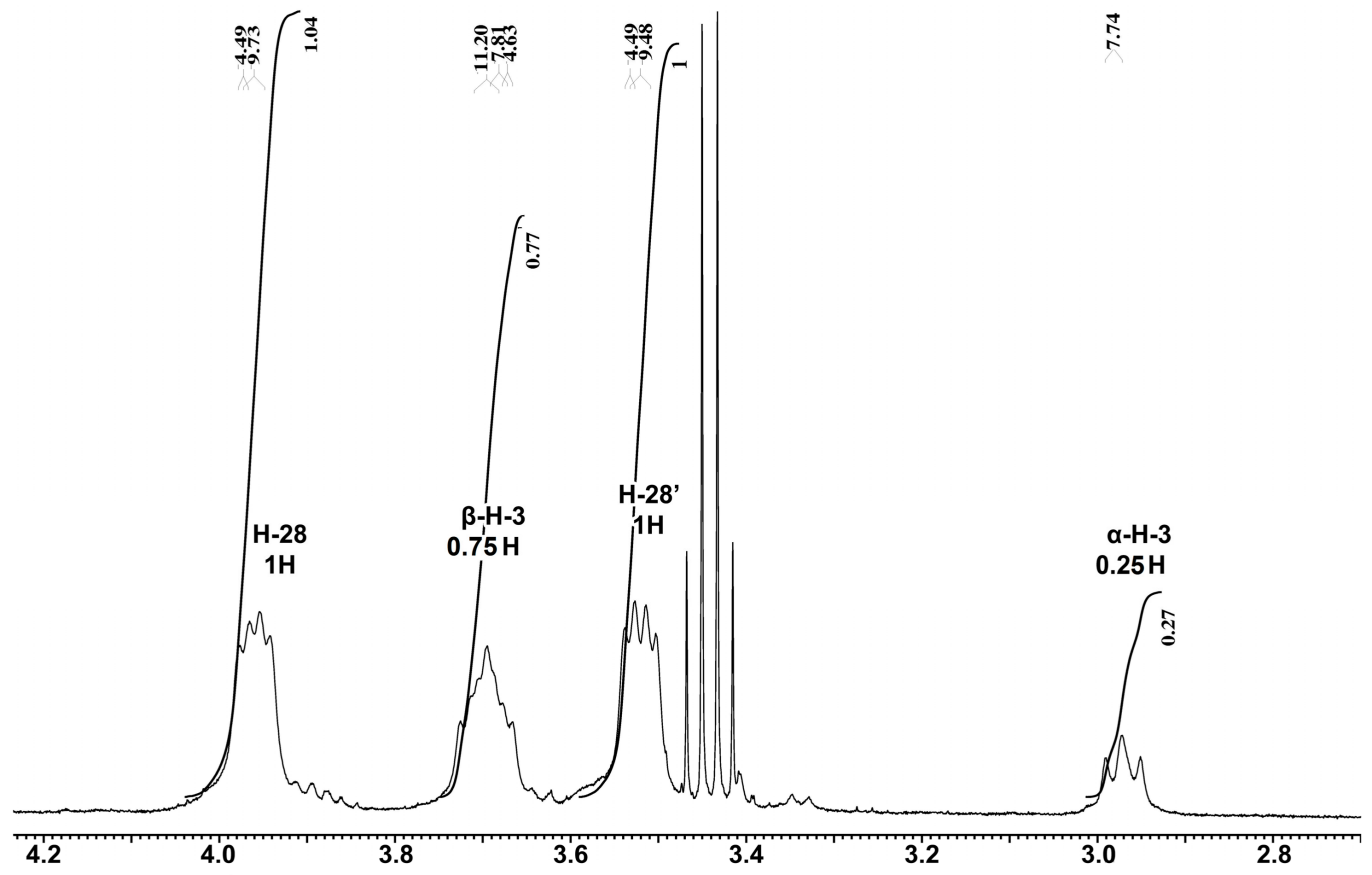
Fragment of the ^1^H-NMR spectrum of BDP-1 stored during one month (DMSO-d_6_, TMS standard, 400 MHz). Note: δ 0.68–1.99 ppm (42H, m, 6CH_3_, (CH_2_)_10,_ (CH)_4_), 2.35-2.42 ppm (1H, m, H-19), 2.97 ppm (0.25H, wide t, α-H-3, *J* = 7.7 Hz), 3.69 ppm (0.75H, ddd, β-H-3 m *J* = 4.6, 7.8, 11.2 Hz), 3.96 ppm (1H, dd, H-28, *J* = 9.7, 4.5 Hz) and 3.52 ppm (H, dd, H-28′, *J* = 9.5, 4.5 Hz), 4.55, 4.69 ppm (2H, two s, H-29), 5.69 (protons in the phosphate groups O-P(O)(OH)_2_, wide blurred s).

The existence of α- and β-epimers of BDP was suggested by the ^1^H-NMR spectra too (Figure 5 and Figure S3). The two epimers had ^1^H-NMR resonance differences of H-3: δ = 2.97 ppm for α-H-3 (wide triplet, *J* = 7.7 Hz) versus δ = 3.69 ppm for β-H-3 (ddd, *J_1_* = 4.6 Hz, *J_2_* = 7.8 Hz, *J_3_* = 11.2 Hz). Similar differences in ^1^H-NMR resonances of the 3-H protons of betulin derivative epimers were shown earlier [23,24]. The ^1^H-NMR resonances of H-28 were observed in the region 3.96 ppm (H-28, dd, *J_1_* = 9.7 Hz, *J_2_* = 4.5 Hz) and 3.52 ppm (H-28′, dd, *J_1_* = 9.5 Hz, *J_2_* = 4.5 Hz).

Readily ethanol soluble BDP-1 was exposed to aging with time and a loss of solubility resulted from BDP-1 structure changes. The BDP-1 samples were thus transformed into BDP-2 with concomitant alcohol solubility loss when the BDP-1 samples were dried at 150 ± 10 °C for 3 h or at room temperature for 3 months, whereas BDP-2 samples were more stable and their properties changed insignificantly.

Considering that the topochemical processes, including “cold crystallization”, epimerization and polymorph transition, processes in solid state of BDP proceed more slowly than in solutions and the epimerization of BDP in the solutions proceeds more easily. The ratio of α- and β-epimers may differ over time. Figure 5 shows the ^1^H-NMR spectrum of a BDP-1 sample stored during one month, where the integral ratio of α-H-3 and β-H-3 signals equals 1:3.

It may be proposed that the transition of the α-epimer to the β-epimer contributes to the change in the solubility and thermoanalytical characteristics of the structural modifications of the BDP. The effect of different structural forms of BDP on the biological activity may be corrected by forming a water-soluble sodium salt.

### 2.2. Water-Soluble Sodium Salt of Betulin-3,28-diphosphate

**Figure 6:**
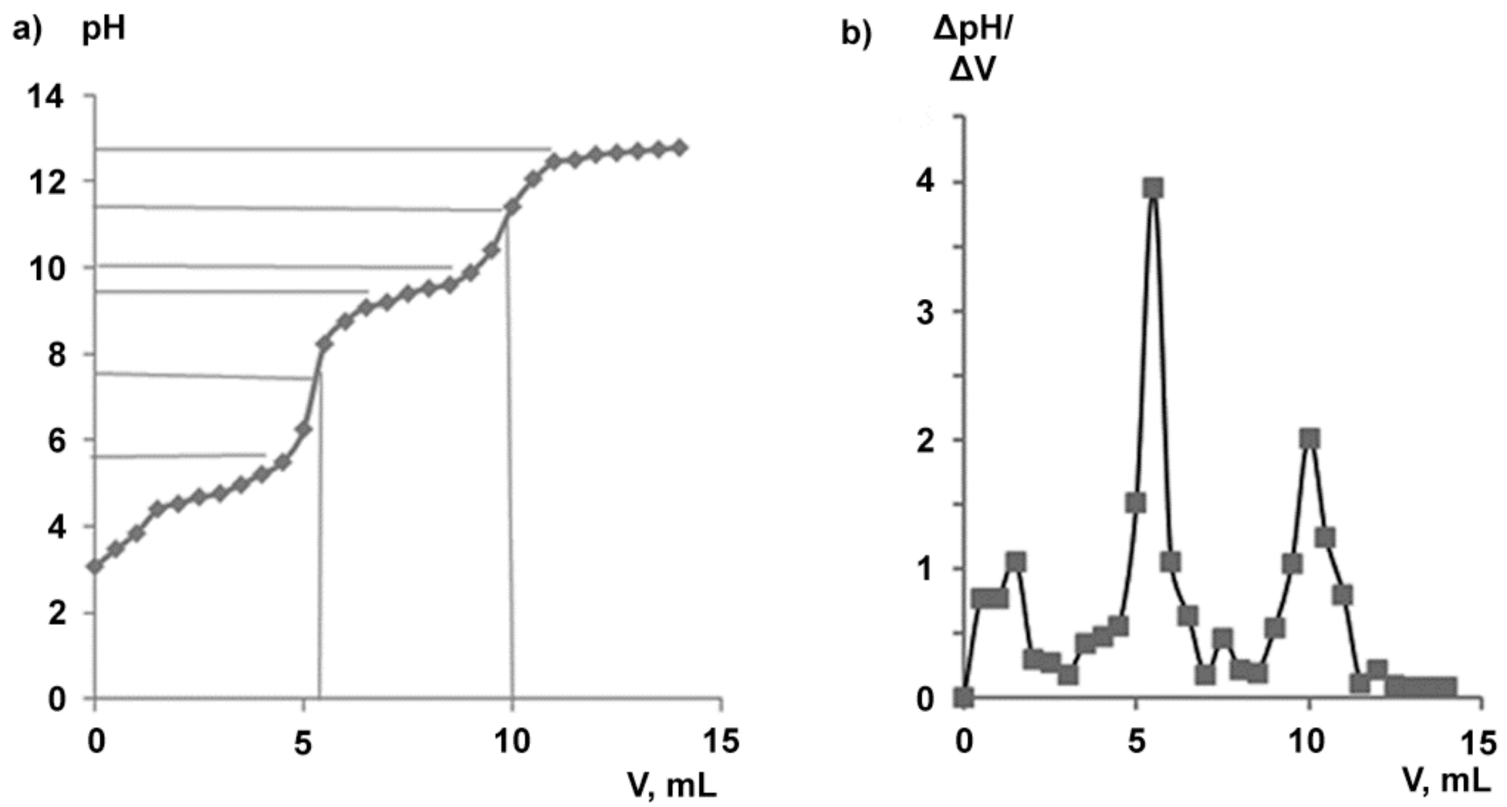
Potentiometric titration of BDP by 0.2 M NaOH aqueous solution: (**a**) pH = f(V); (**b**) differential curve ΔpH/ΔV = f(V), *n* = 8.

The sodium salt of BDP was obtained by adding an aqueous alkaline solution to an ethanol BDP solution. Potentiometric titration of an ethanol solution of BDP-1 by 0.2–4.0 M alkali solutions showed the interaction of BDP and NaOH to proceed in two stages, the stoichiometry of titration being 1:2 and 1:4. Figure 6 shows a typical titration curve pH = f (V) for BDP-1 by aqueous 0.2M NaOH and its differential form ΔpH/ΔV = f (V).

FTIR-spectral data (Figure 1c,d) and ^31^P-NMR spectra (Figure S4) supported the formation of sodium salts of BDP which were precipitated by acetone from aqueous-ethanol solutions at pH 7 and at pH 10. Na-BDP deposited at pH 10 is a crystalline hydrate Na-BDP × 8H_2_O.

**Figure 7:**
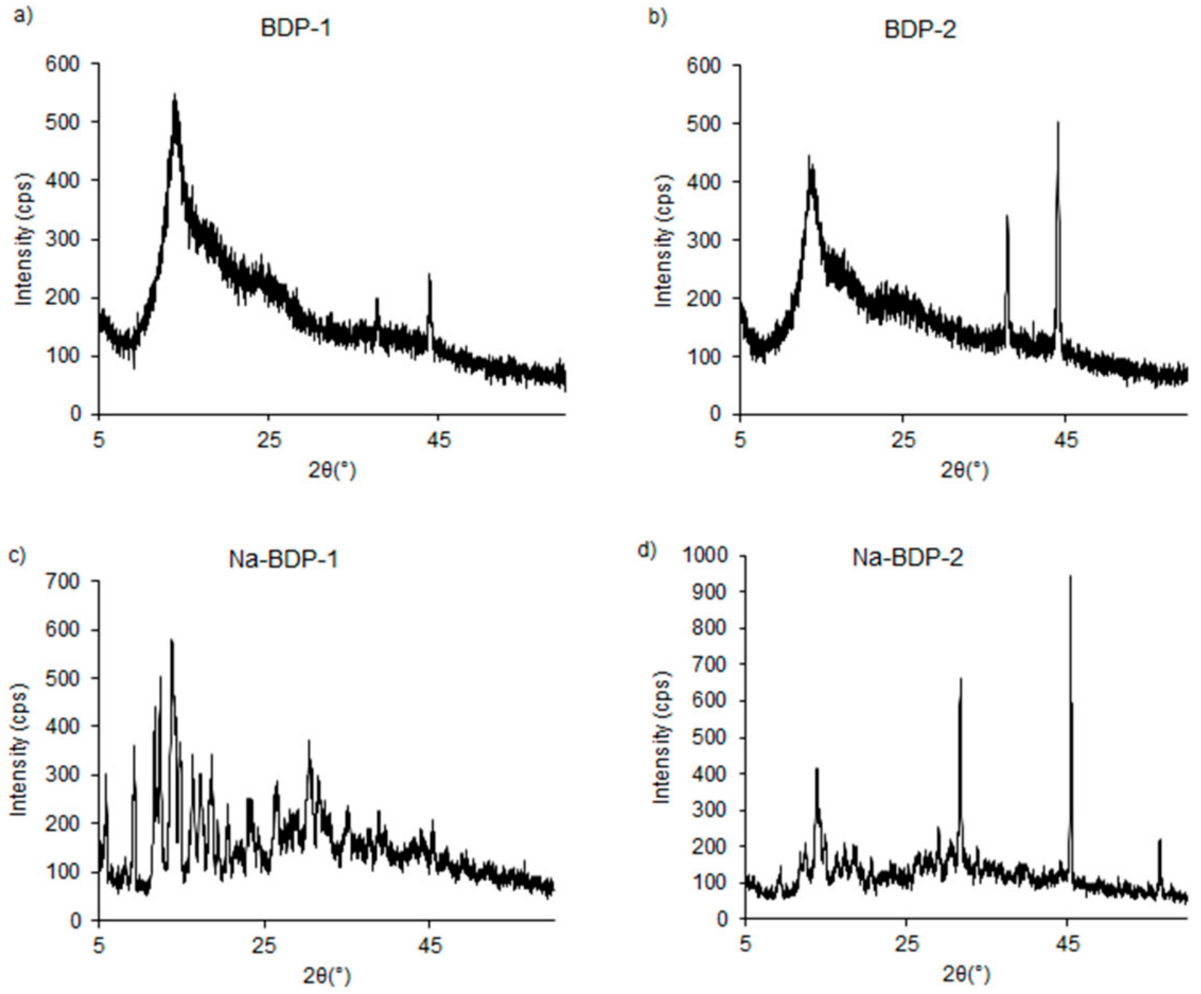
Powder XRD data of the samples BDP-1 (**a**), BDP-2 (**b**), Na-BDP-1 (**c**) and Na-BDP-2 (**d**).

Water-soluble Na-BDP-1 was obtained as needle-like crystals, unlike the poorly crystalline Na-BDP-2, whereas BDP-1 and BDP-2 aged with time and were isolated as an amorphous substance in accordance with powder XRD data (Figure 7). It should be noted that amorphous samples have a bimodal molecular weight distribution, as evidenced by the complex form of the peak at small angles.

**Figure 8:**
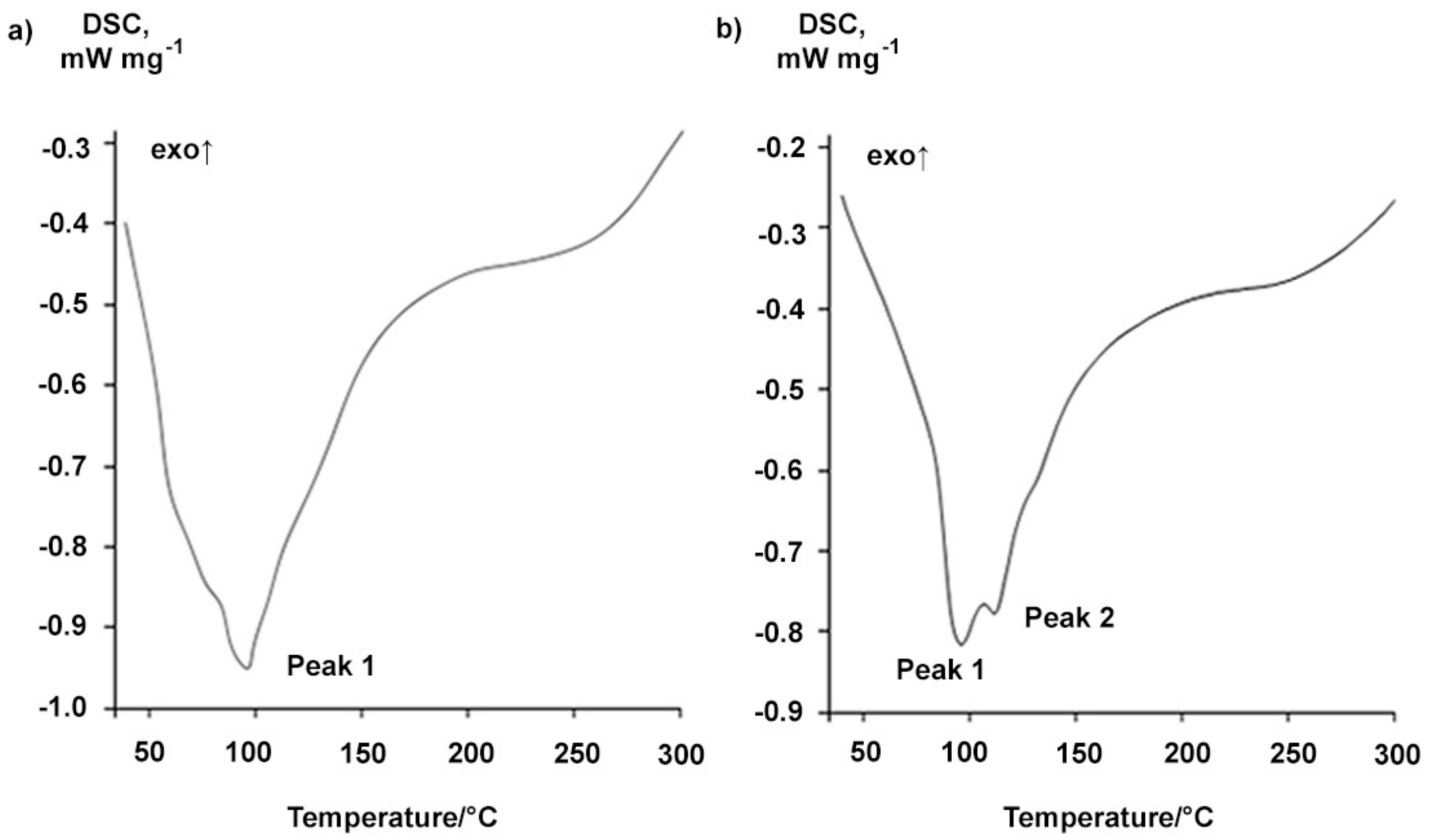
DSC-curves of sodium salt of betulin-3,28-diphosphate: (**a**) Na-BDP-1; (**b**) Na-BDP-2. The number of the experiments repeated was 4.

**Figure 9:**
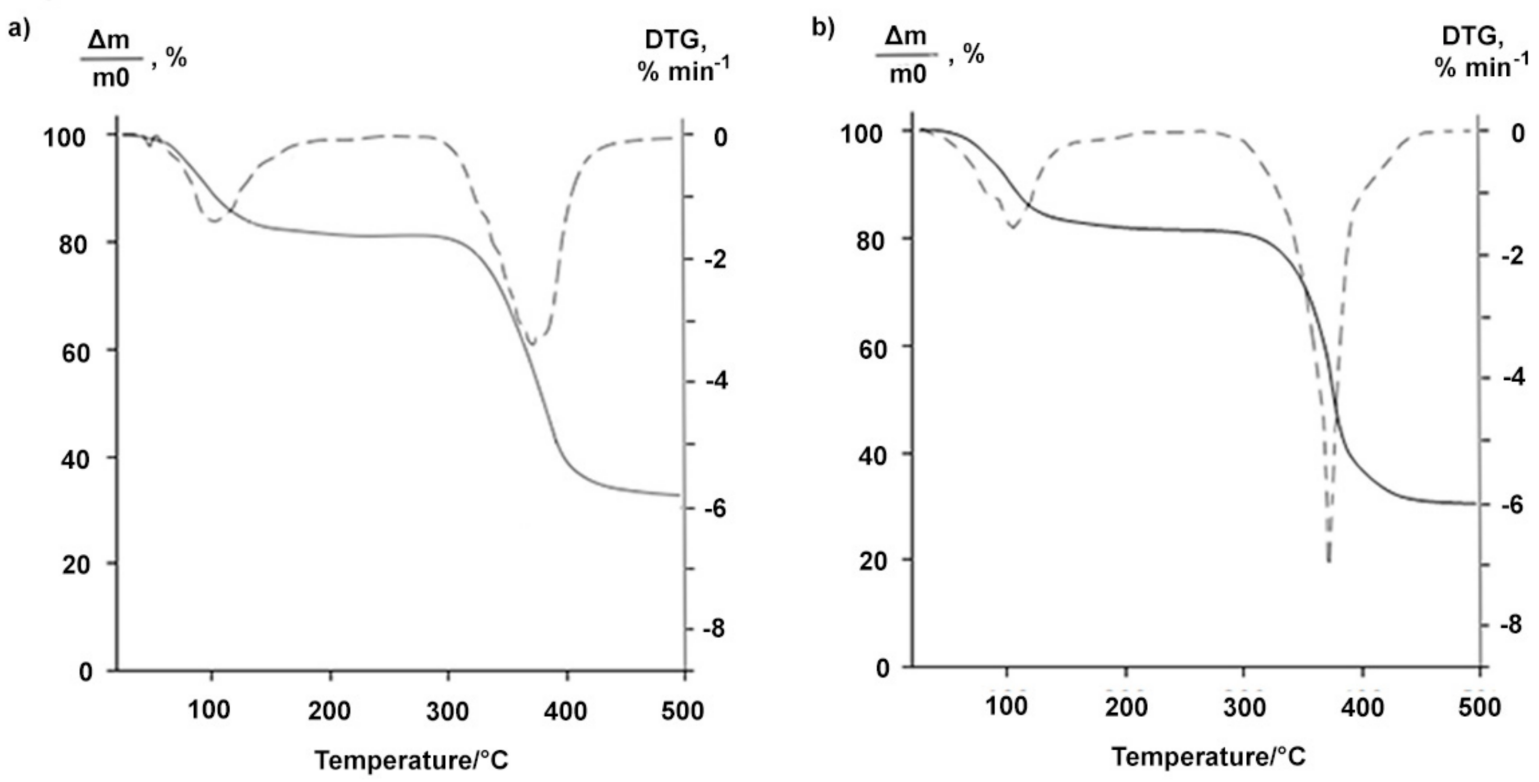
TG (solid line) and DTG (dashed line) curves of betulin-3,28-diphosphate: (**a**) Na-BDP-1; (**b**) Na-BDP-2. The number of the experiments repeated was 4.

The DSC curves of Na-BDP characterize the endo-effect with a maximum at 95–112 °C (Figure 8), probably due to the removal of bound water molecules in Na-BDP hydrates. One can see two stages on the TG curve of the BDP sodium salt (Figure 9), the first stage corresponding to a water loss equal to 16.4%, that is about 7.5 water molecules per the Na-BDP molecule. The effect can be associated with the removal of not only crystallization water from the hydrate structure, but also the occluded water in the crystal lattice or between the solid grains. The process of water loss in Na-BDP crystalline hydrate leads to the loss of its solubility in water during storage.

### 2.3. Biological Activity of Betulin-3,28-diphosphate In Vitro Experiments

We investigated the biological activity of the highly ethanol soluble BDP-1 as its water-soluble sodium salt—Na-BDP. Biochemical indexes, such as antioxidant enzyme activity of catalase and superoxide dismutase SOD, total antioxidant status TAS, lipoperoxidation LPO and malonic dialdehyde MDA level in plasma and erythrocytes in rat blood were evaluated compared to the control. The effect of BDP on energy metabolism was assessed by increasing of the lactate dehydrogenase LDH level in direct and reverse reactions. In addition, the effect on biological activity of poorly soluble in ethanol BDP-2 was studied. In the experiments, a mixture of the BDP forms (BDP-1:BDP-2 = 2:1) was used as the salt complex of BDP with meglumine at the molar ratio 1:4. Table 2 shows the high antioxidant enzymatic activity of BDP, while the effect of BDP-1 exceeds almost twice the activity of the BDP samples mixture. Taking into account the ratio of BDP-1 and BDP-2 in the mixture is 2:1, we can conclude a weak effect of BDP-2 on the enzyme activity. The non-enzymatic activity (LPO decrease) of BDP-1 was also better in comparison with the mixture of BDP-1 and BDP-2 (Table 2).

Moreover, special attention should be given to a stronger BDP effect on BDP-1 energy metabolism in comparison with the mixture. The LDH activity was found to increase fourfold in the direct reaction and in twice the reverse reaction under the action of BDP-1.

## 3. Materials and Methods

### 3.1. Materials

Betulin was isolated from *Betula pendula* bark using the methods in [25]. Phosphorus oxytrichloride (Sigma Aldrich, Moscow, Russia), purified water (resistivity ≥18 MΩ·cm, Millipore, Merck, Darmstadt, Germany).

### 3.2. FTIR Analysis

FTIR spectra in the 400–4000 cm^−1^ range were measured by an IR Prestige-21 FTIR spectrometer (Shimadzu, Kyoto, Japan) equipped with a KBr beam splitter. For performing these measurements, a pellet from well-dried KBr was prepared by a standard cold pressing method. Resolution was 0.5 cm^−1^. The number of scans was 45.

### 3.3. NMR Analysis

^13^С-, ^1^H-, ^31^P-NMR spectra were recorded at 101, 400 and 202,46 MHz, respectively, on a JNM-ECX400NMR-spectrometer (Jeol Ltd., Tokyo, Japan), using as solvents DMSO-*d*_6_, CDCl_3_, or D_2_O.

### 3.4. Differential Scanning Calorimetry

To investigate the thermal behavior and to measure the heat capacities of BDP under study within the temperature range from t = 20 to 300 °C, we used a DSC 204 F1 Phoenix differential scanning calorimeter with μ-sensor (Netzsch−Gerätebau, Selb, Germany). The calorimeter was calibrated and tested against melting of standard calibration set (indium, bismuth, zinc, tin, biphenyl, mercury, cesium chloride, and potassium nitrate) [26]. It was established that the apparatus and the measurement procedure enabled to measure the temperature of phase transitions with the standard uncertainty u(*T*) = 0.5 °C, and the enthalpies of transitions with the combined expanded uncertainty U_c,r_ (∆*H^o^_tr_*) = 0.01. The temperatures and the enthalpies of phase transitions were evaluated according to the standard Netzsch Proteus Software (Selb, Germany) procedure. The heat capacity was determined by the ratio, sapphire being used as a standard reference sample. The technique to determine the temperatures and the enthalpies of transitions is described in detail [27,28] and in Netzsch Proteus Software. The heating rate was 5 °C min^−1^; it being measured in argon atmosphere.

### 3.5. Thermogravimetric Analysis

The TG curves were recorded in the 20–500 °C range, on a TG 209 F1 Iris TG analyzer (Netzsch−Gerätebau) using open ceramic/aluminum sample pans (sample mass 4–16 mg), under dynamic argon atmosphere (25 mL∙min^−1^) and heating rate of 5 °C min^−1^.

### 3.6. Powder X-ray Diffraction Analysis

Powder X-ray diffraction patterns were obtained using Shimadzu X-ray diffractometer XRD-6000 at 295(2) K with Cu Kα radiation (λ = 1.5418 Å), in the Bragg-Brentano reflection geometry. The samples were collected in the 2*θ* range between 5–50° with steps of 0.026° and 100 s of step size, using a scan speed (°/s) of 0.067335. On the X-ray diffraction patterns of amorphous samples, there are diffraction peaks at 37.5° and 44.0° referring to the cuvette material.

### 3.7. Synthetic Procedures

#### 3.7.1. Synthesis of Betulin-3,28-diphosphate (BDP, 3β,28-diphosphate-lup-20(29)-ene)

Phosphorus oxytrichloride (7.56 mL, 81.6 mmol; Sigma Aldrich, Moscow, Russia, 99%) in dioxane (60 mL) was added dropwise to a solution of betulin (6.0 g, 13.56 mmol) in a mixture of dioxane (120 mL) and pyridine (7.08 mL, 81.6 mmol) at 10–20 °C into a three-necked flask. The reaction mixture was stirred for 24 h at room temperature and then treated by water and ice mixture (1000 g) in the beaker. White precipitate was filtered off and washed by water many times. A wet sediment contained 3–25% of water that corresponds to the hydrate of betulin-3,28-diphosphate × H_2_O, where x = 1–9 (7.90 g, 94% as dried product): mp: 147–148 °C (BDP-1), 156–160 °C (BDP-2); UV (EtOH): *λ*_max_ (log *ε*) 256 (2.64) nm; FTIR (KBr): ν_max_ 3421, 2331, 2342, 1641-1700, 1240, 1031, 973, 501 cm^−1^; ^1^H- NMR (DMSO-*d*_6_, 400 MHz) δ 0.68–1.99 (42H, m, 6CH_3_, (CH_2_)_10_, (CH)_4_), 2.35–2.42 (1H, m, H-19), 2.97 (0.25H, wide t, α-H-3, *J* = 7.7 Hz), 3.69 (0.75H, ddd, β-H-3 m, *J* = 4.6, 7.8, 11.2 Hz), 3.96 (1H, dd, H-28, *J* = 9.7, 4.5 Hz) and 3.52 (H, dd, H-28′, *J* = 9.5, 4.5 Hz), 4.55, 4.69 (2H, two s, H-29), 5.69 (protons in the phosphate groups O-P(O)(OH)_2_, wide blurred s); ^13^C-NMR (DMSO-*d*_6_, 101 MHz) *δ* 149.93 (C, C-20), 109.92 (CH_2_, C-29), 82.96 (CH, =CHOH), 63.22 (CH_2_, CH_2_OH), 54.90 (CH, C-5), 49.71 (CH, C-9), 48.11 (CH, C-19), 47.26 (CH, C-18), 46.75 (C, C-17), 42.30 (C, C-14), 40.48 (C, C-8), 38.57 (C, C-4), 38.35 (CH_2_, C-1), 37.99 (C, C-10), 37.07 (CH, C-13), 36.57 (CH_2_, C-7), 33.79 (CH_2_, C-22), 29.11 (CH_2_, C-21), 28.96 (CH_2_, C-16), 28.17 (CH_3_, C-23), 27.90 (CH_2_, C-2), 26.57 (CH_2_, C-15), 24.83 (CH_2_, C-12), 20.43 (CH_2_, C-11), 18.84 (CH_3_, C-30), 18.01 (CH_2_, C-6), 16.14 (CH_3_, C-26), 15.91 (CH_3_, C-24), 15.70 (CH_3_, C-25), 14.56 (CH_3_, C-27); ^31^P-NMR (DMSO-*d*_6_, 202.46 MHz) δ −0.4 (d, *J* = 8.2 Hz, phosphoric acid residue at C-3β), 0.48 ppm (t, *J* = 4.6 Hz, phosphoric acid residue at C-28).

In the ^13^C-NMR spectrum the signals of the C-28 and C-3 atoms of BDP (60 and 80 ppm, respectively) were shifted by 3 ppm in comparison with botulin, which is characteristic for phosphoric acid esters.

The ^31^P-NMR spectra of BDP in DMSO-d_6_ were obtained in the presence of Ph_3_P (δ = −6.0 ppm) and H_3_PO_4_ (δ = −0.14 ppm) which was added just before recording the spectrum at 30 °C. The phosphoric acid residue at C-3β of BDP in the spectrum without decoupling of the protons was recorded as a doublet (δ = −0.4 ppm), while the coupling constant J_H-P_ ~ 8 Hz is typical for CH-O-P (Figure S1). The phosphoric acid residue at C-28 of BDP is represented as a triplet at δ = +0.48 ppm, with a coupling constant J_H-P_ ~ 4.6 Hz that is characteristic of the CH_2_-O-P fragment of phosphatidic acids (alkylacyl glycerophosphates), where the signal (δ = +0.55 ppm, the coupling constant J_H-P_ ~ 6.9 Hz) corresponds to the -CH_2_-O-P(O)(OH)_2_ fragment [29].

Phosphorus content determined by spectrophotometric analysis [30] was equal to 9.59–10.32% (Calc. content for C_30_H_52_O_6_P_2_ is equal to 10.30%).

BDP assay was performed by reversed phase HPLC analysis: 210 nm, 40 °C, mobile phase A30%-B70% *v/v* (A—acetonitrile (grade 0), B—buffer solution of KH_2_PO_4_, pH = 6.36), flow 1.0 mL∙min^−1^. The retention time is 5.19 min.

BDP-1 and BDP-2 separation. The wet BDP crude (9 g) was dissolved in ethanol (600 mL) and the insoluble part (BDP-2) was filtered off. BDP-1 was isolated by precipitation from BDP ethanol solution with acetone, and BDP-1 was recrystallized from ethanol.

#### 3.7.2. Synthesis of the Sodium Salt of Betulin-3,28-diphosphate

The sodium salt of betulin-3,28-diphosphate (Na-BDP) was obtained by adding 0.2–4.0 M aqueous alkaline (Na_2_CO_3_ or NaOH) solutions to aqueous or ethanol BDP solutions. Na-BDP-1 and Na-BDP-2 were obtained from BDP-1 and BDP-2, respectively. Na-BDP-1 in the form of Na-BDP 8H_2_O crystalline hydrate (water content 16.80–17.27%) was obtained by adding 4 M NaOH solution to alcohol solution of BDP-1. UV (H_2_0): λ_max_ (log ε) 256 (2.62) nm; FTIR (KBr) ν_max_ 3384, 2330, 1641–1700, 1375, 1089, 975, 513 cm^−1^; ^31^P-NMR (D_2_O, 202.46 MHz) δ 4.25, 4.31 (d, *J* = 8.4 Hz, phosphoric acid residue at C-3β), 5.47 ppm (t, phosphoric acid residue at C-28). Sodium salt of BDP was obtained as Na_2_-BDP at pH 7 and as Na_4_-BDP at pH 10. Figure S4 shows the ^31^P-NMR spectrum of a Na_4_-BDP sample. Calculated phosphorus content for C_30_H_48_O_8_P_2_Na_4_ is equal to 9.09%, while the phosphorus content found for Na_4_-BDP at pH 10 was equal to 8.94% after drying. Calculated phosphorus content for C_30_H_50_O_8_P_2_Na_2_ is equal to 9.60%, while the phosphorus content found for Na_2_-BDP at pH 7 was equal to 9.54% after drying.

### 3.8. Biological Activity In Vitro

Biological activity in vitro was studied using blood stabilized with sodium citrate (1:9). Antioxidant properties were estimated with respect to the malonic dialdehyde (MDA) level in plasma and erythrocytes according to Uchiyama and Mihara technique [31] and superoxide dismutase (SOD, EC 1.15.1.1) activity. The energy metabolism in erythrocytes was studied using the catalytic activity of lactate dehydrogenase (LDH, EC 1.1.1.27) in the direct (substrate—50 mM sodium lactate) and reverse (substrate—23 mM sodium pyruvate) reaction [32,33].

The study as presented was approved by the Local Ethics Committee of Privolzhsky Research Medical University, Russian Federation (Protocol No.2 dated 20 February 2016).

## 4. Conclusions

Betulin-3,28-diphosphate samples obtained from betulin and POCl_3_ in dioxane in the presence of pyridine as HCl acceptor in molar ratio of betulin:POCl_3_:pyridine equal to 1:6:6 at 0–25 °C during 24 h have two main structural forms, BDP-1 and BDP-2, which differ in ethanol solubility, melting point, FTIR spectra and thermoanalytical characteristics.

Readily water-soluble sodium salts of BDP obtained by adding alkaline (NaOH or Na_2_CO_3_) solution to BDP solution differed too: Na-BDP-1 was obtained as needle-like crystals unlike poorly crystalline Na-BDP-2.

Thermoanalytical methods (DSC and TG) are convenient methods to study BDP structural forms. The exo-effects on the DSC curves at 193 ± 8 °C can be used as the criteria to distinguish between BDP samples: −19.7 ± 0.2 kJ∙mol^−1^ for BDP-1 and −70.5 ± 0.7 kJ∙mol^−1^ for BDP-2 (sample reprecipitated by acetone). Another distinctive sign of BDP-1 is the presence of a first stage on the TG curve, which characterizes the loss of mass Δm/m_0_ of 5–7%, to 150 °C, whereas for BDP-2 samples this stage is absent. The combination of BDP properties such as solubility, melting temperature, FTIR spectral characteristics, exo-effects on DSC curves and TG analysis data make it possible to definitely characterize type 1 (BDP-1) and type 2 (BDP-2). The endo-effects of Na-BDP on DSC curves are probably related to the removal of water from the structure of the initially formed hydrate.

The most probable reason for the transition of BDP-1 to BDP-2 are different topochemical processes, e.g., “structural reconstruction” leading to amorphous substances in accordance with powder XRD data.

Therefore, the use of BDP as a water-soluble potential active pharmaceutical ingredient is desirable in the form of Na-BDP × 8H_2_O crystal hydrate, that enables to ensure a satisfactory solubility of the preparation and, accordingly, its high biological activity estimated in vitro experiments on rats.

## Figures and Tables

**Table 1:** Thermophysical characteristics of the samples of betulin-3,28-diphosphate ^1^.

Type of BDP	Sample	Melting Point, °С	Peak 1	Peak 2			Peak 3		
			T_peak_, °С	T_onset_, °С	T_peak_, °С	Exo-Effect ΔH, kJ∙mol^−1^	T_onset_, °С	T_peak_, °С	Exo-Effect ΔH, kJ∙mol^−1^
**1**	Readily soluble in ethanol (up to 10%)	148	85.3 ± 0.5	151.2 ± 0.5	155.9 ± 0.5	−15.5 ± 0.2	183.5 ± 0.5	200.8 ± 0.5	−19.7 ± 0.2
**2**	After boiling water treatment during 1 h	157	77.0 ± 0.5	143.2 ± 0.5	161.0 ± 0.5	−10.0 ± 0.1	171.0 ± 0.5	185.1 ± 0.5	−80.4 ± 0.8
	Synthesis from **Na-BDP ^2^**	156	90.8 ± 0.5	137.5 ± 0.5	148.5 ± 0.5	−21.4 ± 0.2	164.2 ± 0.5	187.3 ± 0.5	−67.1 ± 0.7
	Reprecipitation by acetone	160	94.1 ± 0.5	161.3 ± 0.5	172.8 ± 0.5	−23.5 ± 0.2	185.9 ± 0.5	197.3 ± 0.5	−70.5 ± 0.7

^1^ Standard uncertainty of temperature u(*T*) = 0.5 °C. Сombined expanded uncertainty for the enthalpies of transitions U_c,r_ (∆*H^o^_tr_*) is 0.01. The number of experiments repeated was 4. ^2^ BDP was obtained from BDP sodium salt by the action of an aqueous solution of 0.1 M aqueous HCl.

**Table 2:** Biochemical parameters of rat blood under the influence of BDP aqueous solutions in the dose 2 μg∙mL^−1^ ^1^.

Parameters	% of Control	
	BDP-1 as Sodium Salt	Mixture of BDP-1 and BDP-2 (2:1) ^2^
Catalase	299.0 ± 13.6	170.5 ± 3.5
SOD	334.9 ± 2.0	165.2 ± 2.5
MDA_plasma_	98.9 ± 13.0	115.07 ± 0.2
MDA_erythrocyte_	57.0 ± 8.0	83.1 ± 3.7
LDH_direct_	423.6 ± 7.0	271.2 ± 6.5
LDH_reverse_	236.6 ± 4.0	110.0 ± 3.0
LPO_plasma_	92.8 ± 0.9	101.2 ± 0.7
LPO_erythrocyte_	68.6 ± 0.5	96.8 ± 1.4
TAS	117.6 ± 1.9	102.7 ± 4.0

^1^ The number of the repeat experiments was three. ^2^ A mixture of BDP-1 and BDP-2 was dissolved in water in the presence of meglumine.

## Supplementary Materials

The following are available online at http://www.mdpi.com/1420-3049/23/5/1175/s1, Figure S1: ^31^P-NMR spectrum of BDP (DMSO-d_6_, standard Ph_3_P in the presence of H_3_PO_4_), Figure S2: ^31^P-NMR spectra of BDP, DMSO-d_6_. a) BDP-1 sample, standard Met_3_P; b) BDP-1 in the presence of H_3_PO_4_, standard Ph_3_P (δ = -5.93 ppm) and H_3_PO_4_ (δ = -0.4 ppm); c) BDP-2 sample, standard Met_3_P, Figure S3: ^1^H-NMR spectrum of BDP. DMSO-d_6_, standard TMS, 400 MHz, Figure S4: ^31^P-NMR spectrum of Na-BDP. D_2_O, standard H_3_PO_4_.
