# Supplementary material for: Betulin-3,28-diphosphate. Physico-Chemical Properties and In Vitro Biological Activity Experiments
Source: Molecules. 2018 May 14;23(5):1175. doi: 10.3390/molecules23051175 (PMC6099732; doi:10.3390/molecules23051175)
Supplement: Supplementary file 1 [file molecules-23-01175-s001.pdf]

List of changes

We marked our changes in the text by green color.

Page 1

M1

All the names are correct.

We would like to add affiliations department names for Alexander V. Knyazev (Department of Solid State Chemistry) and Alexey V. Markin (Department of Physical Chemistry).
We corrected some abbreviations of author's names.

M2

We would like to keep e-mail melnikovanb@gmail.com

M3

Bold is not necessary in this case. We removed it in whole paper.

M4

DSC is differential scanning calorimetry.
TG is thermogravimetric analysis.

Page 2

M5

We defined DSC/TG earlier.

Page 3

M6

We would like to move the Figure 10 to Supplementary Materials as Figure S1.

Page 5

M7

We would prefer not to insert this space.

Page 7

M8

"n" should not be italic.

M9

We confirm the correction.

Page 9

M10

We confirm the correction.

3.1 Materials

Betulin was isolated from Betula Pendula bark using the methods in [25. Kuznetsova, S.A.; Skvortsova, G.P.; Maliar, I.N.; Skurydina, E.S.; Veselova, O.F. Extraction of betulin from birch bark and study of its physico-chemical and pharmacological properties. Russ. J. Bioorganic Chem. 2014, 40, 742–747]. Phosphorus oxytrichloride (Sigma Aldrich, Moscow, Russia), purified water (resistivity ≥18 MΩ·cm, Millipore, Merck, Darmstadt, Germany).

M11

Shimadzu, Kyoto, Japan

M12

Jeol Ltd., Tokyo, Japan

M13
Netzsch–Gerätebau, Selb, Germany

M14
We corrected the order of references.

M15
We would prefer not to insert this space.

M16
Netzsch Proteus Software (Selb, Germany)

Page 10

M17
Phosphorus oxytrichloride (7.56 mL, 81.6 mmol; Sigma Aldrich, Moscow, Russia, 99%)

M18
We confirm the correction.

Page 11

M19
Italic is not necessary.

M20
Ethical code number is correct.

Page 12

We would like to change "Na-BDP·8H_2O" to "Na-BDP × 8H_2O".

M21

The following are available online at www.mdpi.com/xxx/s1, Figure S1: ^{31}P-NMR spectrum of BDP (DMSO-d_6, standard Ph_3P), Figure S2: ^{31}P-NMR spectra of BDP, DMSO-d_6. a) BDP-1 sample, standard Met_3P; b) BDP-1 in the presence of H_3PO_4, standard Ph_3P (δ = -5.93 ppm) and H_3PO_4 (δ = -0.4 ppm); c) BDP-2 sample, standard Met_3P, Figure S3: ^{1}H-NMR spectrum of BDP. DMSO-d_6, standard TMS, 400 MHz, Figure S4: ^{31}P-NMR spectrum of Na-BDP. D_2O, standard H_3PO_4.

We don't know the correct link of our Supplementary Materials. Please, add this link, if you don't mind.

M22
We confirm the correction.

M23
Funding: This research received no external funding.

M24
We would like to remove section Acknowledgments, if it is possible.

Page 13

M25
Patent RU 2,243,233, 27 December 2004.

M26
Patent US 3,764,616, 9 October 1973.

<u>Page 14</u>
M27
Sample Availability information is correct.
